# A Blue-Purple Pigment-Producing Bacterium Isolated from the Vezelka River in the City of Belgorod

**DOI:** 10.3390/microorganisms9010102

**Published:** 2021-01-05

**Authors:** Nikita S. Lyakhovchenko, Tatiana N. Abashina, Valentina N. Polivtseva, Vladislav Yu. Senchenkov, Daniil A. Pribylov, Anna A. Chepurina, Ilja A. Nikishin, Alina A. Avakova, Michael A. Goyanov, Elizaveta D. Gubina, Daria A. Churikova, Alexander A. Sirotin, Nataliya E. Suzina, Inna P. Solyanikova

**Affiliations:** 1 Federal State Autonomous Educational Institution of Higher Education, Belgorod National Research University, 308015 Belgorod, Russia; nikitkibullmail@gmail.com (N.S.L.); vladiksenchencov@gmail.com (V.Y.S.); daniil.pribylov@yandex.ru (D.A.P.); post0765@yandex.ru (A.A.C.); ilaynikishin@gmail.com (I.A.N.); avakova1200@gmail.com (A.A.A.);666mihai999@gmail.com (M.A.G.); dml1xngubina@yandex.ru (E.D.G.); daria.tchurikova@yandex.ru (D.A.C.); sirotin19402702@mail.ru (A.A.S.); 2 G.K. Skryabin Institute of Biochemistry and Physiology of Microorganisms, Pushchino Center for Biological Research of the Russian Academy of Sciences, Pushchino, 142290 Moscow, Russia; tanica@rambler.ru (T.N.A.); kaistia@gmail.com (V.N.P.); suzina_nataliya@rambler.ru (N.E.S.)

**Keywords:** *Janthinobacterium* sp., identification, violacein, biotechnological significance

## Abstract

Violacein is a biotechnologically significant secondary metabolite due to its antibacterial, antifungal, and other properties. Isolation, research, and identification of violacein producing strains are of interest for the development of biotechnological processes, in order to enhance the biosynthesis of this compound. The purpose of the present work was to study the properties of a newly isolated bacterium capable of synthesizing blue-purple pigment. An aboriginal bacterium was isolated from the coastal zone of the Vezelka River in the city of Belgorod. Based on chemical and spectrophotometric studies of the crude ethanol extract, the pigment was identified as violacein, and the isolate was assigned to the group of violacein-forming bacteria, which includes bacteria of the genera *Chromobacterium*, *Iodobacter*, *Janthinobacterium*, *Duganella*, *Collimonas*, and *Massilia*. Based on cultural, morphological, tinctorial, physiological, and biochemical properties, as well as analysis of the 16S rRNA gene sequence, the new isolated strain was assigned to the genus *Janthinobacterium*. The isolated strain is capable of suppressing the growth of a number of fungal and bacterial phytopathogens. For representatives of the genus *Janthinobacterium*, their inhibitory influence on cyanobacteria was shown for the first time.

## 1. Introduction

Bacterial cells produce a huge number of biologically active compounds, including vitamins, amino acids, antibiotics, and enzymes [1]. At present, the biotechnological significance of one more group of secondary metabolites of bacteria, pigments, has been estimated [2]. The ability to synthesize pigments that differ in chemical composition, color, and solubility is characteristic of many microorganisms [3].

**Figure 1:**
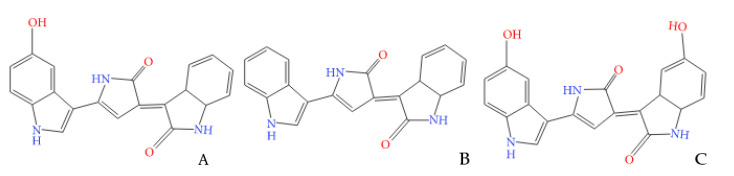
The structure of the violacein (**A**), deoxyviolacein (**B**), and oxyviolacein (**C**) molecules [6].

The role of pigments for microorganisms may be to participate in respiration processes, to protect against ultraviolet radiation. In addition, some microorganisms synthesize pigments that have antibacterial and/or antifungal properties. The formation of secondary metabolites by microorganisms, providing their antagonistic effects, is an adaptation to the conditions of existence in the microbial community along with other forms of interaction both within the consortium and for other systems associated with it. The phenomenon of antagonism among microorganisms is widely used in medicine and agriculture. Live antagonist microbes are used in medical practice to combat dysbacteriosis and candidomycosis, which sometimes occur with the use of broad-spectrum antibiotics, for therapy and prevention of various infectious diseases. Antagonism between microorganisms attracts the attention of scientists and agricultural workers for its use in the fight against phytopathogenic organisms that cause considerable harm to agricultural production [4]. Thus, bacteria of the genera *Chromobacterium*, *Iodobacter*, *Janthobacterium*, *Duganella*, *Collimonas*, and *Massilia* are able to form a purple pigment-violacein or (3- (1,2-dihydro- (5-hydroxy-1H-indol-3-yl) -2-oxo-3H-pyrrol-3-ylidene) -1,3-dihydro-2H-indol-2-one) (Figure 1A), which has important biological activity (antitumor, antiviral, antibacterial, antifungal, antiprotozoal, and antiparasitic activity) and significant pharmacological potential [5].

It is known that the formation of violacein is due to the mechanisms of the sense of quorum. Based on the fact that the pigment is visible to the naked eye, violacein-synthesizing bacteria act as a convenient model in studies of the effect of various molecules on the sense of quorum. The violacein biosynthetic pathway involves five genes, namely *vioA*, *vioB*, *vioC*, *vioD*, and *vioE*, identified in bacteria by the condensation of two L-tryptophan molecules [6].

By the creation of a composition of violacein and an antagonist *Bdellovibrio bacteriovorus* HD100, active against a polymicrobial system consisting of *Staphylococcus aureus*, *Acinetobacter baumannii*, *Bacillus cereus*, and *Klebsiella pneumoniae*, it was shown that violacein can be used in complex systems to control undesired microflora. The antagonistic efficacy of violacein and *B. bacteriovorus* HD100 was 19% and 68%, respectively, whereas the joint activity was 99.98%. At the same time, violacein had no effect on *B. bacteriovorus* HD100 [7].

Based on the literature data, violacein (0.9 μM) had a stimulating effect on the productivity of recombinant immunoglobulin by Chinese hamster ovary cells by 37.6%. The study of the authors reveals another biotechnological significance of the pigment, in addition to the antagonistic properties described earlier, so violacein can be used as a stimulant in the biosynthesis of monoclonal antibodies [8].

One of the interesting directions to use the ability of microorganisms to synthesize violacein turned out to be environmental biotesting. Thus, a biosensor was constructed that is sensitive to high concentrations of Pb (II) compounds with pigmentation as an output signal. According to the mechanism indicated by the authors, violacein is formed as a result of the conversion of two molecules of endogenous L-tryptophan under the influence of five proteins, which are transcribed and translated as a single polycistronic unit under induction of Pb (II) [9].

Deoxyviolacein (Figure 1B) is produced by wild strains of bacteria as a byproduct of violacein biosynthesis. It differs from violacein in that it contains 1 oxygen atom less in the 6th position of the indole ring. Violacein extracts produced by wild strains typically contain up to 10% deoxyviolacein. Oxyviolacein is another structural analogue of violacein, with an additional oxygen atom at 20th position of the indole ring. Oxyviolacein (Figure 1C) can be obtained using exogenous 5-hydroxy-1-tryptophan (5-HTP) as a precursor due to the lack of a high substrate specificity of enzymes involved in violacein biosynthesis [10].

A purple bacterium was isolated from the coastal zone of the Vezelka River in the center of Belgorod. The purpose of this work was to study the properties of this bacterium and assess its ability to control the growth of other microorganisms living in the aquatic ecosystem.

## 2. Materials and Methods

### 2.1. Strain 

The native strain of the pigment-forming bacterium was isolated from the riparian zone of the Vezelka River in the central region of the city of Belgorod by inoculating serial dilutions of river water samples on LB nutrient medium [11]. The initial crops of river water samples were incubated at 4 °C. Colonies that had a blue-purple color were selected. After making sure of the purity of the culture named JF4, microscopy and cytochemical studies were performed. The strain was deposited in the All-Russian collection of microorganisms under the number VKM B-3515.

### 2.2. Morphology, Culture Media, and Growth Conditions

To determine the conditions for optimal bacterial growth, a complete liquid medium IBPM 5/5 was used. This medium contained (g/L): soy extract—30, casein hydrolyzate—5, yeast extract—1, and aminopeptide—60 mL/L (pH 7.2), agar (20 g/L) was added to prepare a solid medium. The temperature optimum was determined in the range 4–45 °C. The halotolerance of the isolates was determined by growing the cultures in media with NaCl content in the range from 0 to 10%. The growth of cultures in both cases was evaluated by the optical density determined at a wavelength of 590 nm in a 10 mm cuvette using a UV-1800 spectrophotometer instrument. (Shimadzu, Kyoto, Japan). Each variant of the experiment was carried out in triplicate. 

The oxygen requirement of the JF4 isolate was determined by injection into agar. For this, 3% peptone agar (g/100 mL: peptone—3; Na_2_HPO_4_—0.2; NaCl—0.3; agar—2) was poured into 8 mL tubes and sterilized. The cells were inoculated into solidified agar. By the character of growth in the thickness of the medium, the attitude of the culture to O_2_ was judged [11].

Calculation of the diameter of bacterial colonies used the root mean square, since it is used for average traits, expressed in terms of volume, area, or diameters of a circle [12]:

$$S=\sqrt{\frac{\sum V^{2}}{n}};$$

where *S* is the root mean square, *V* is the date, and *n* is the number of measurements.

The number of measurements was 100 values of the colony diameter.

Gram staining was performed using the standard method. The negative Burri method was used to detect the capsules. The motility of bacterial cells was determined by the “hanging drop” method [13].

### 2.3. Microscopy

#### 2.3.1. Light Microscopy 

Bacteria cells were examined under a Nikon Eclipse Ci microscope (Nikon, Tokyo, Japan) equipped with a camera ProgRes Speed XT core5 (Jenoptik, Jena, Germany) and an immersion oil lens with phase contrast with magnification 100×. Colonies were examined by a stereoscopic microscope, MICROMED II (St-Petersburg, Russia). 

#### 2.3.2. Electron Microscopy of Ultrathin Sections

Cells were prefixed with 1.5% (*v/v*) glutaraldehyde solution in 0.05 M cacodylate buffer (pH 7.2) at 4 °C for 1 h. After three washings with the same buffer, the material was additionally fixed with 1% OsO_4_ in 0.05 M cacodylate buffer at 20 °C for 3 h. After dehydration, the material was embedded into epoxy resin Epon 812. Ultrathin sections were made on an Ultramicrotome REICHERT-JUNG ULTRACUT (Wien, Austria). The sections were mounted on copper grids covered with a Formvar film, contrasted with uranyl acetate (3% solution in 70% ethanol) for 30 min, and then stained with lead citrate [14] at 20 °C for 4–5 min. The sections were examined in a JEM-1400 electron microscope (JEOL, Tokyo, Japan) at an 80 kV accelerating voltage.

### 2.4. Biochemical Characteristics 

To determine the induction of catalase in the JF4 bacterium, a 3% solution of hydrogen peroxide was applied to its colony. The presence of catalase was judged by the formation of oxygen bubbles [11].

The proteolytic activity of JF4 bacteria was determined by its ability to hydrolyze casein. To detect it, we used milk agar, consisting of sterile defatted by centrifugation at 8000× *g*, milk, and 2% agar. Casein hydrolysis is detected in the zone of clarification of the medium near the culture stroke [11].

The lipolytic activity of the JF4 bacterium was studied by inoculation on a peptone nutrient medium containing (g/L) Tween80—10 mL; peptone—10; NaCl—5; CaCl_2_·H_2_O—0.1 [6]. During the hydrolysis of Tween-80, the products formed react with calcium chloride. As a result, turbidity is formed around the colonies [11].

The presence of urease was determined on a nutrient medium, in which the carbon source was carbamide (composition (g/L): (NH_2_)_2_CO—5.0; K_2_HPO_4_—0.5; Na-citrate—5.0). Carbamide was added after autoclaving. A litmus paper soaked in sterile water was placed under the plug. The presence of urease was judged by the release of ammonia, which changes the color of the litmus test to blue. In addition, the presence of ammonia in the culture liquid was determined using commercial Nessler’s reagent (OOO Ural Chemical Plant, Perm, Russia). The reaction of ammonium sulfate with sodium hydroxide can act as a control, as a result of which ammonia is released [11].

The ability of a culture to form indole on a nutrient medium with peptone was determined using the Salkovsky reaction (1 mL of 0.2% KNO_3_ was added to the culture fluid obtained by centrifugation of liquid nutrient medium samples (using an Eppendorf 5418 R centrifuge, Hamburg, Germany) with the JF4 bacterium, which was cultivated for 24 h. Then, a few drops of strong sulfuric acid were added. If indole is present in the medium, a complex compound is formed, which stains the medium red) [11].

The phosphatase activity of the JF4 culture was determined by growing the isolate on a nutrient medium containing inorganic calcium phosphate. By the formation of discoloration zones near the sowing, the ability of the bacteria to dissolve phosphate was judged [11].

The enzyme activity and the spectrum of assimilated substrates for the JF4 strain were determined using commercial Kits API 20E (bioMerieux, Craponne, France) according to the protocol.

To determine the spectrum of substrates utilized by bacteria, the API 50 CH Kit (bioMerieux, Craponne, France), designed to study the carbohydrate metabolism of microorganisms, was used according to the protocol.

### 2.5. Resistance to Antibiotics

To analyze the resistance of the strains to antibiotics, 100 μL of the studied culture in the exponential growth stage was applied to Petri dishes with 5/5 solid medium and evenly distributed over the entire surface of the dish using the spatula. The antibiotic discs (CJSC NICF, St-Petersburg, Russia) were laid out on top of the test culture at an equal distance from each other and from the edges of the Petri dish. The results were evaluated after 24 h of culture at 25 °C by the presence of a lysis zone around the disc. If such zone was absent, the strain was resistance to a given concentration of antibiotic.

### 2.6. The Ability of the JF4 Strain to Control the Growth of Various Microorganisms 

The antibacterial activity of the secondary metabolites of the strain JF4 was investigated by the formation of the lysis zone of the test culture. For this study strain JF4 was preliminarily grown in a IBPM 5/5 liquid medium. Bacteria in the exponential growth phase was precipitated by centrifugation (10,000× *g*, 10 min) and the supernatant containing the products of the secondary metabolism and traces of bacterial cells was used in the experiment to determine the antibacterial activity. Then, on Petri dishes with a solid medium IBPM 5/5, 100 μL of the test culture in the exponential growth stage was applied and evenly distributed over the entire surface of the dish with the spatula. Sterile filter paper disks were laid on top of the test culture under study, and 5 μL of the supernatant was applied to them. The results were evaluated after 24 h of culture at 25 °C by the presence of a lysis zone around the paper disk. The presence of such zone means the activity of the studied strain in relation to the strain of the test culture.

As test cultures, we used 22 strains of Gram-positive and Gram-negative bacteria of such genera as *Pseudomonas*, *Escherichia*, *Staphilococcus*, *Erwinia*, *Alcaligenes*, *Bacillus*, *Micrococcus*, *Deinococcus*, and *Achromobacter* received from both the All-Russian collection of microorganisms (VKM) and from the working collection of the authors.

The antifungal activity of the strain using the example of the fungus *Alternaria brassicola* F-1864 was investigated by the method of perpendicular streaks, which is based on perpendicular sowing of a strip of a test culture of mold to a strip of isolate JF4 in a nutrient medium Sabouraud [11].

The anti-algal activity of the JF4 culture against cyanobacteria was judged by the formation of lysis zones of the test culture around a paper disk (on a nutrient medium for BG_N_-11 cyanobacteria [11]) saturated with JF4 in the logarithmic growth phase.

### 2.7. Phylogenetic Analysis

Genomic DNA was isolated from cells using the Fungal/Bacterial DNA Kit (Zymo Research, USA) according to the manufacturer’s recommendation. The 16S rRNA gene was amplified by PCR using primers universal for 16S rRNA prokaryotes: 27f (5′-AGAGTTTGATCCTGGCTCAG3′) and 1492r (5′-TACGGYTACCTTGTTACGACTT3′) [15]. The amplified DNA was purified using the Zymoclean Gel DNA Recovery Kit (ZymoResearch, Irvine, CA, USA). Sequencing of PCR DNA fragments was performed on an Applied Biosystems 3130 Genetic Analyzer automatic sequencer.

Primary phylogenetic screening of the obtained sequences was performed using the BLAST program (http://www.ncbi.nlm.nih.gov/blast) in the EzBioCloud database (www.ezbiocloud.net). For phylogenetic analysis, 16S rRNA gene sequences were taken from the GenBank database (www.ncbi.nlm.nih.gov). The nucleotide sequences of the 16S rRNA gene obtained for the JF4 strain were manually aligned with the sequences of reference strains of the nearest microorganisms. Phylogenetic tree constructed using partial 16S rRNA gene sequences by the neighbor-joining method with a bootstrap test of 1000 replicates was performed using MEGA 6.0. 

### 2.8. Pigment Extraction

The pigment was extracted with 96% ethanol. For this, after culturing bacteria in a liquid nutrient medium consisting of peptone (3%) at 25 °C for 72 h, the cell biomass was precipitated by centrifugation at 14,000× *g* for 10 min. After that, the culture supernatant was taken and the precipitate was poured with 96% ethanol. It was shaken for 30 min, centrifuged again, and the crude ethanol extract of the blue-purple pigment was collected. Re-extraction was performed. The absorption maximum of the crude ethanol extract of the pigment was determined spectrophotometrically at the wavelength range λ = 350–700 nm [3] using a Multiskan GO spectrophotometer (Thermo Scientific, Waltham, MA, USA). In addition, the pigment was determined by adding 10% sulfuric acid dropwise to the crude ethanol extract until the solution appeared green [16].

## 3. Results and Discussion

### 3.1. Cell Morphology

**Figure 2:**
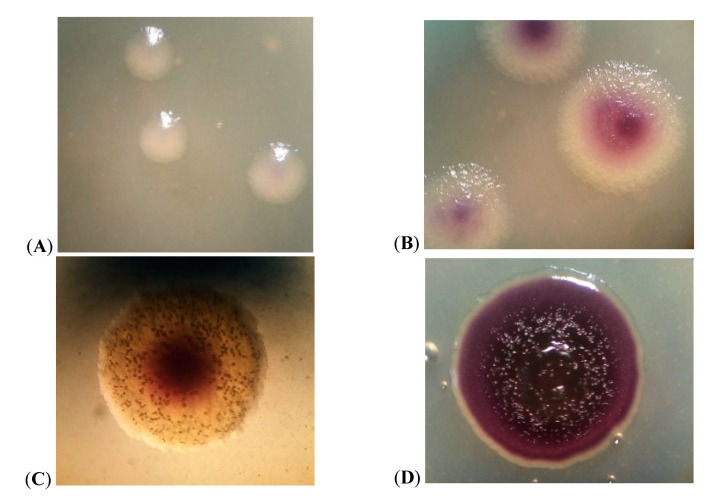
JF4 colonies on 3% peptone agar at 25 °C at: (**A**) the second day of incubation; (**B**) the third day of incubation; (**C**) the fourth day of incubation; (**D**) the seventh day of incubation.

The formation of colonies of strain JF4 on 3% peptone agar occurred on the second day of incubation at 25 °C. Colonies were colorless, opaque, smooth, convex, slightly pigmented in the center (Figure 2A). On the third day of incubation, a noticeable blue-purple pigmentation appeared, the colonies were granular with a scalloped edge (Figure 2B). On the fourth day of incubation, the colony pigmentation became more intense, the structure was granular, and the edge was uneven (Figure 2C). The pigmentation of the JF4 on the seventh day of incubation was pronounced blue-purple, the colonies were rough, coarse-grained, dull, and round with a wavy ridge along the edge (Figure 2D), crater-like profile. Isolation of metabolite crystals was observed, which were clearly visible in the lumen of the stereoscopic microscope MICROMED II (Figure 2C). 

The root mean square diameter of the colonies on the fourth day of incubation was 1.93 mm, which slightly increased with further exposure. In a liquid nutrient medium, on the second day of incubation, a pigmented ring of culture overgrowth was formed at the interface.

According to the literature, representatives of bacteria of the genus *Chromobacterium* on solid nutrient media forms oily, smooth, or rough pigmented colonies, depending on the species (Table 1), and in liquid media form a purple ring along the vessel wall at the surface of the liquid [17,18,19,20,21,22,23,24,25,26,27,28,29,30]. For bacteria of the genus *Iodobacter*, the formation of thin, wrinkled, spreading colonies with a diameter of 1 cm or more is characteristic on nutrient media; oily consistency (Table 1); pigmentation starts from the center of the colony; on a liquid nutrient medium, representatives form a purple ring, like bacteria of the genus *Chromobacterum* [31,32,33,34,35,36]. In turn, colonies of representatives of the genus *Janthinobacterium* are characterized by the formation on solid nutrient media of slightly convex in the center, rounded, purple color (Table 1). A purple ring is also formed on the surface of the liquid medium along the vessel wall [17,37,38,39,40,41]. Representatives of the genus *Duganella*, forming a blue-purple pigment, form wrinkled, slightly leathery colonies on agar nutrient medium (Table 1), while a flocculent sediment is observed in a liquid one [16,42,43,44]. Literature data on the cultural properties of the genera *Collimonas* and *Massilia* under consideration are not given in the sources [45,46,47,48,49,50]. Thus, based on the literature data, the cultural properties of JF4 in a liquid nutrient medium are similar to those of *Chromobacterium* sp., *Iodobacter* sp., and *Janthinobacterium* sp., as they form a purple ring along the vessel wall at the interface. However, the cultural properties of JF4 colonies on agar culture medium correspond to the genus *Janthinobacterium* with a bulge in the center and a round shape. At the same time, JF4 is characterized by the formation of a viscous, slightly leathery consistency, which is characteristic of representatives of the genus *Duganella*.

During the study of colonies of the JF4 population, it was noticed that the intensity of pigmentation changes during incubation, which may be associated with the density of cells in the colony.

**Figure 3:**
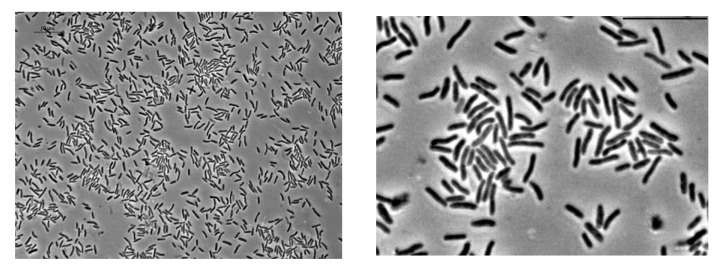
Phase contrast microscopy. Cells of the JF4 strain in the logarithmic growth phase. Scale bar—10 μm.

It was revealed that the JF4 bacteria are represented by aerobic Gram-negative motile rods, located singly, in pairs, or in short chains (Figure 3). Analysis of ultrathin cell sections showed that the cells have a cell wall structure typical for Gram-negative bacteria. The cell wall was characterized by the presence of an outer membrane lined by a thin underlying murein layer. Cells are 2–2.5 × 0.6–0.7 μm in size, rod-shaped with pointed ends. Analysis of ultrathin sections suggest that the pigment is presumably visible (taking into account all preparative procedures during the preparation of ultrathin sections) in the form of accumulations of fine fibrillar-granular material bound or near to the cell surface.

**Figure 4:**
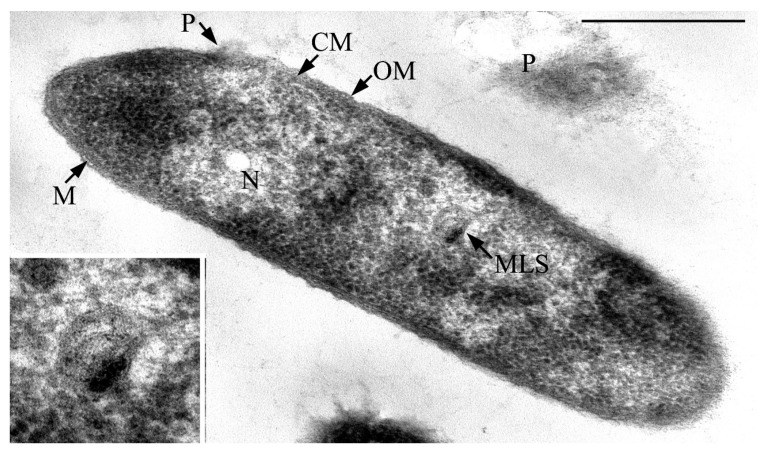
Transmission electron microscopy. Ultrathin section of JF4 strain cell. The inset in the lower left corner of the figure is an enlarged fragment of the cell cytoplasm with a membrane-like structure (MLS). Designations: CM, cytoplasmic membrane; OM, outer membrane; M, murein; MLS, membrane-like structures; N, nucleoid; P, residues of modified after preparation pigment. Scale bar = 0.5 μm.

Membrane-like structures curled into a ball were revealed in the central part of the cytoplasm of JF4 cells. These structures are very similar to mesosomes. No intracellular inclusions were found on ultrathin sections of the cells by the type of reserve inclusions (Figure 4).

### 3.2. Physiological and Biochemical Characteristics of the Strain

The study of the physiological and biochemical characteristics of the isolate allows us to conclude that they are catalase-positive, form cytochrome oxidase, and hydrolyze casein. On a nutrient medium with peptone (3%), they do not form indole, but produce ammonia. Hydrogen sulfide is not produced. They do not synthesize urease, lipase, and lecithinase; they hydrolyze casein, but not starch. The strain is not capable of liquefying gelatin, but it is capable of utilizing citrates. The test for phosphatase activity was positive for the strain (Table 1). The analysis did not reveal the activity of β-galactosidase, arginine dihydrolase, lysine decarboxylase, ornithine decarboxylase, and tryptophanedaminase. The strain does not form acetoin. The bacteria of the isolated JF4 strain are similar in their characteristics to representatives of the genus *Chromobacterium*, namely, Gram staining, motility, shape, ends, and arrangement of cells. They are oxidase positive and indole negative. However, JF4 differs from *Chromobacterium* sp. in such important features as the attitude to oxygen, the ability to hydrolyze gelatin, and lecithinase activity [17,18,19,20,21,22,23,24,25,26,27,28,29,30]. The similarity of JF4 with representatives of the genus *Iodobacter* is manifested in Gram staining, cell shape and arrangement, absence of oxidase, lysine decarboxylase, ornithine decarboxylase, and β-galactosidase, it does not hydrolyze starch [31,32,33,34,35,36]. At the same time, the JF4 strain is distinguished by lecithinase activity, oxygen ratio, and motility (Table 1). The isolated strain is similar to representatives of the genus *Janthinobacterium* in the shape and arrangement of cells, Gram staining, in relation to oxygen-aerobic, lack of the ability to form indole and lecithinase, the ability to use citrate, and the presence of catalase and oxidase. When cultured in liquid peptone, they form ammonia. The test for phosphatase activity was positive for strains [17,37,38,39,40,41]. The investigated isolate is similar to pigment-forming bacteria of the genus *Duganella* in Gram reaction, cell shape and arrangement, motility, attitude to oxygen, ability to form catalase, to hydrolyze casein, and inability to form indole (Table 1). However, representatives of the genus *Duganella* hydrolyze starch and gelatin and form lipase (Table 1) [16,42,43,44]. Thus, the JF4 isolate differs greatly from the representatives of the genus *Duganella* in its characteristics. At the same time, it is similar in its features to bacteria of the genera *Collimonas* and *Massilia* only in the shape of cells, motility, Gram staining, the presence of catalase, and the relation to oxygen (Table 1). Other signs differ or are not given in the literature [45,46,47,48,49,50].

The results of the analysis of data on the study of carbohydrate metabolism of the studied strain using API 50 CH tests showed that the JF4 strain is able to utilize a wide range of substrates (Table 1), namely glycerol, L-arabinose, D-ribose, D-xylose, D-galactose, D-glucose, D-fructose, D-mannose, inositol, D-mannitol, D-sorbitol, N-glucosamine, esculin, D-maltose, D-sucrose, xylitol, D-lyxose, D-arabitol, and potassium 2-ketogluconate. In turn, erythritol, D-arabinose, L-xylose, D-adonitol, methyl-βD-xylopyranoside, D-sorbose, L-rhamnose, dulcitol, methyl-αD-mannopyranoside, methyl-αD-glucopyranoside, amygdalin, arbutin, salicin, D-cellobiose, D-lactose (bovine), D-melibiose, D-trehalose, inulin, D-melezitose, D-raffinose, amidone, glycogen, gentiobiose, D-turanose, D-tagatose, D-fucose, L -fucose, L-arabitol, potassium gluconate, and potassium 5-ketogluconate are not utilized (Table 1). When comparing the obtained data with the literature data for the genus *Chromobacterium*, a similarity was revealed in the ratio of D-mannose and salicin, while the ability to utilize glycerol, L-arabinose, D-xylose, D-galactose, esculin, D-sucrose, and D-trehalose was different (Table 1). Other information was not found in literature sources [17,18,19,20,21,22,23,24,25,26,27,28,29,30]. Data on the ability to utilize substances for bacteria of the genus *Iodobacter* showed a similar result (Table 1) [31,32,33,34,35,36]. The JF4 isolate by its features turned out to be similar to those given in the literature for bacteria of the genus *Janthinobacterium* in relation to the ability to utilize citrates, L-arabinose, D-xylose, D-galactose, D-glucose, D-fructose, D-mannose, inositol, D-mannitol, D-sorbitol, D-maltose, D-sucrose, D-trehalose, D-melicytosis, D-raffinose, and starch, while they differ in relation to inulin and D-lactose (Table 1). Other investigated signs are not given in the sources [17,37,38,39,40,41]. Common to JF4 and representatives of *Duganella* sp. turned out to be the ability to utilize erythrol, D-xylose, D-galactose, D-glucose, D-mannitol, N-acetylglucosamine, and D-maltose, but they are different in terms of the ability to utilize glycerol, D-fructose, salicin, D-cellobiose, D-lactose (Table 1). Most of the studied JF4 characters are not presented in the literature for *Duganella* sp. [16,42,43,44]. Based on the literature data, for bacteria of the genus *Collimonas* and *Massilia*, many of the determined properties of JF4 have not been given, however, there is evidence that the isolate and representatives of *Collimonas* sp. Are similar in the ability to utilize inositol, but different in respect of D-trehalose (Table 1), while JF4 are similar to *Massilia* sp. the ability to utilize L-arabinose, D-xylose, and D-maltose, but different in relation to citrates, D-galactose, D-glucose, D-fructose, L-rhamnose, D-cellobiose, D-lactose, and D-sucrose [45,46,47,48,49,50].

### 3.3. Growth Characteristics of JF4 Strain

The cells of the JF4 strain grow in the temperature range of 4–28 °C with an optimum at 20 °C, which made it possible to classify it as a facultatively psychrophilic organism (Table 1). Among the bacteria capable of forming a blue-purple pigment at 4 °C, there are representatives of *Iodobacter* sp. and *Janthinobacterium* sp., while the optimum temperature range varies [31,32,33,34,35,36,37,38,39,40,41].

The study of the halotolerance of the JF4 isolate revealed its ability to grow in the presence of NaCl in a concentration of up to 2% in the medium; no growth was observed with an increase in the concentration (Table 1). At NaCl concentrations above 2%, representatives of the genus *Massilia* are able to grow (3%, but not at 4%). For bacteria, *Iodobacter* sp., *Janthinobacterium* sp., and *Duganella* sp. are unable to grow at NaCl concentrations above 1%, but for *Chromobacterium* sp. and *Collimonas* sp., data on the ability to grow at high concentrations are not given in the literature [16,17,18,19,20,21,22,23,24,25,26,27,28,29,30,31,32,33,34,35,36,37,38,39,40,41,42,43,44,45,46].

### 3.4. Antimicrobial Activity

It was found that secondary metabolites of the strain JF4 expressed antibacterial activity only against two Gram-negative bacteria of the genus *Pseudomonas* and *Achromobacter* and Gram-positive bacteria of the genera *Micrococcus*. The rest of the tested test cultures were found to be insensitive to the action of the JF4 strain (Table 2). Nevertheless, the cells of the JF4 strain themselves had an inhibitory effect on the development of colonies of such organisms as *Alternaria* and *Aspergillus*. In addition, they suppressed the growth of cyanobacteria isolated from the same source as the studied strain. This activity of cells indicates their mode of survival and competition with other strains in their natural habitat. Considering that violacein-forming bacteria were isolated not only from water, but also from soil samples, the ability to control the growth of other strains via the synthesis of secondary intermediates can be an effective adaptation for maintaining the population size.

**Figure 5:**
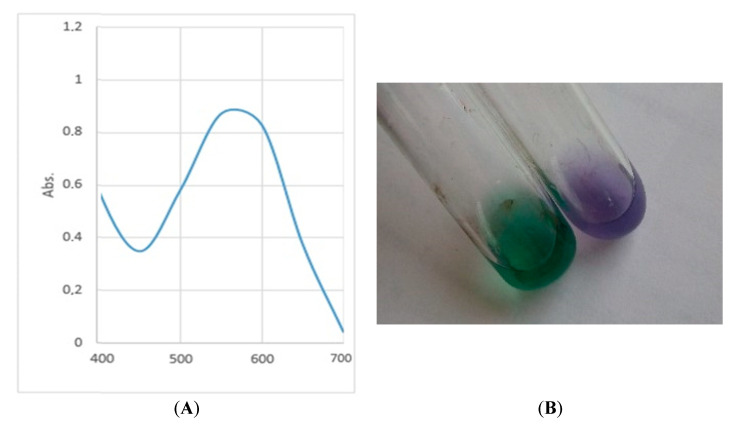
Properties of ethanol extract pigment: (**A**) Spectra of the crude ethanol extract of the pigment strain JF4 in the range from 400 to 700 nm. The absorption maximum was found at 575 nm. (**B**) Qualitative reaction of the crude ethanol extract of the pigment strain JF4 with strong sulfuric acid.

A spectrophotometric study of the crude ethanol extract of the blue-purple pigment showed that the absorption maximum of the solution was at a wavelength (λ) of 575 nm (Figure 5A). After adding strong sulfuric acid, the color of the solution turned from purple to green (Figure 5B). Based on the data obtained, it can be assumed that the pigment is similar to violacein in its properties [3]. Thus, the studied bacterium JF4 may belong to the violacein-producing group.

### 3.5. Antibiotic Resistance 

In the course of the work, the JF4 strain was checked for the resistance to antibiotics (Table 3). For this purpose, 38 commercial antibiotics belonging to various groups were used. It was found that the JF4 strain is sensitive to most antibiotics used in the work.

**Figure 6:**
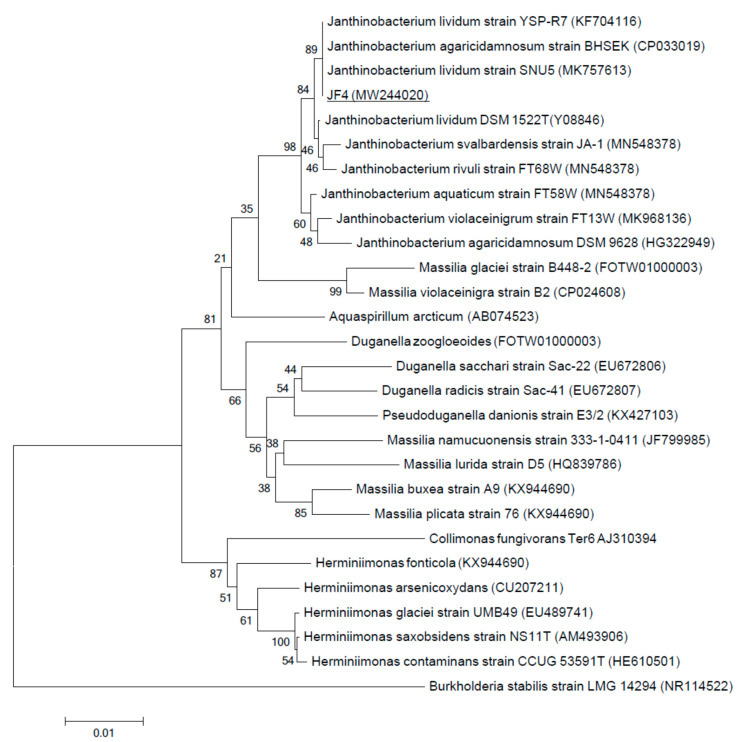
Neighbor-joining tree based on the 16S rRNA gene sequences of strain JF4. Bar, 0.01 substitutions per nucleotide position. Bootstrap percentages >50% based on 1000 replications are shown at the branch points. *Burkholderia stabilis* strain LMG 14294 was taken as the outgroup.

Phylogenetic identification of the JF4 strain by 16S rRNA showed that it belongs to the species *Janthinobacterium lividum* with more than 99% identity (Figure 6). The JF4 strain is most closely related to bacteria *J. lividum* and *J. agaricidamnosum.* The JF4 strain is similar to the bacterium *J. lividum* in the ability to use maltose, D-mannose, inositol, inosine, L-arabinose, D-galactose, D-mannitol, glycerol, D-xylose, sorbitol, xylitol, D-lyxose, 2-ketogluconate, N-acetylglucosamine, esculin, and citrate. The bacterium *J. agaricidamnosum* and JF4 are similar in their ability to utilize glycerol, D-fucose, and citrate. Like *J. lividum*, JF4 was found to be incapable of using gentiobiose, D-trehalose, and turanose. Similar to *J. agaricidamnosum*, JF4 is unable to use D-raffinose, L-rhamnose, D-arabinose, arbutin, salicin, cellobiose, inulin, and maltose [39]. 

The 16S rRNA sequence of the isolated strain was deposited in the GenBank *Janthinobacterium* sp. JF4 MW244020.

## 4. Conclusions

Violacein-synthesized bacteria are described in the literature. They belong to various genera of the class of β-proteobacteria of the families *Neisseriaceae* and *Oxalobacteriaceae*. All of them are characterized by gram-negative motile rod-shaped aerobic or facultatively anaerobic bacteria that produced violacein from tryptophan, differing in their individual properties (Table 1). Nevertheless, there is still very little data on both the biochemical properties of these bacteria and the molecular biological characteristics. As a result of the present studies, it was found that the *Janthinobacterium* sp. strain JF4, isolated from water, synthesizes a pigment similar to violacein, which has a pronounced antimicrobial effect, so that the culture can compete for development in different ecosystems. Furthermore, the low values of the temperatures optimal for growth allow this strain to remain in a metabolically active state for a long time in comparison with less psychrophilic microorganisms. The JF4 strain is capable of growing at low temperatures, most likely does not carry antibiotic resistance plasmids, since it exhibits sensitivity to a large number of widely-used antibiotics, along with the ability to control the growth of various bacterial and fungal phytopathogenic strain, these properties make it possible to characterize the isolated strain as an native culture which promotes sustainable development of ecosystems. During the study of the individual properties of the JF4 isolate, it was revealed that it has activity against cyanobacteria. One of the interesting features is the almost complete absence of antimicrobial activity in the cultural medium of this strain. On the contrary, cells being in contact with other cells exhibit a pronounced ability to control the growth of these organisms. Undoubtedly, this may be due to the specificity of the synthesis of violacein or other metabolites exhibiting this inhibitory effect. When studying the literature data, no mention of such properties of bacteria of the genus *Janthinobacterium* was found. Thus, it can be assumed that this property was shown for the first time. 

## Figures and Tables

**Table 1:** Differentiating characteristics of violacein-producing bacteria.

Properties	Study Cultures						
	JF4	*Chromobacterium* [17,18,19,20,21,22,23,24,25,26,27,28,29,30]	*Iodobacter* [31,32,33,34,35,36]	*Janthinobacterium* [17,37,38,39,40,41]	*Duganella* [16,42,43,44]	*Collimonas* [45,46]	*Massilia* [47,48,49,50]
pigment formation	+	+	+	+	+	+	+
Colonies:							
round scalloped	+	-	-	-	-	ND	ND
round with a roller on the edge	+	-	-	-	-	ND	ND
colony profile	crater-shaped	ND	ND	ND	ND	ND	ND
surface of colonies	wavy	ND	ND	ND	ND	ND	ND
smooth		+	-	-	-	ND	ND
rough	+	+	-	-	+	ND	ND
convex	+	-	-	+	+	ND	ND
structure	coarse-grained	ND	ND	ND	ND	ND	ND
oily consistency	-	+	+	-	-	ND	ND
viscous	+	-	-	-	+	ND	ND
root mean square diameter of colonies, mm	1.93	ND	10	ND	ND	ND	ND
Cell shape:							
rod-shaped	+	+	+	+	+	+	+
globular bacteria	-	-	-	-	-	-	-
straight	+	+	+	+	+	+	-
slightly curved	+	-	-	+	+	+	-
Situated:							
singly	+	+	+	+	ND	-	ND
in pairs	+	+	+	+	ND	+	ND
in chains	+	+	+	+	ND	-	ND
Gram stain	Gram-	Gram-	Gram-	Gram-	Gram-	Gram-	Gram-
dispute formation	-	-	-	-	-	-	-
motility	+	+	+	+	+	+	+
Relationship to O_2_:							
obligate aerobic	+	-	-	+	+	+	+
microaerophilic	-	-	-	-	-	-	-
facultatively anaerobic	-	+	+	-	-	-	-
obligate anaerobic	-	-	-	-	-	-	-
growth ability with 4 °C	+	-	+	+	-	-	-
optimum growth temperature	20–25 °C	30–35 °C	25 °C	25 °C	28–37 °C	20–30 °C	ND
optimal pH	7–8	7–8	4–5	7–8	7–7.5	6.5	ND
growth at 1% NaCl	+	+	+	+	-	ND	+
growth at 3% NaCl	-	ND		-	-	ND	+
growth at 4% NaCl	-	ND	-	-	-	ND	-
growth at 6% or more, NaCl	-	ND	-	-	-	ND	ND
requirements for growth factors	-	-	-	-	-	ND	ND
oxidase	-	+	-	+	+	+	-
catalase	+	+	ND	+	+	- or weak	+
indole	-	-	-	-	-	ND	ND
ureaza	-	ND	-	ND	+	ND	-
lecithinase	-	+	+	-		ND	ND
lipase	-	-	-	-	+	+	ND
β-galactosidase	-	ND	-	ND	-	ND	
arginine dihydrolase	-	ND	ND	ND	-	ND	+
lysine decarboxylase	-	ND	-	ND	ND	ND	ND
ornithine decarboxylase	-	ND	-	ND	ND	ND	ND
tryptophanedaminase	-	ND	ND	ND	ND	ND	ND
acetoin formation (Voges–Proskauer reaction)	-	ND	ND	ND	ND	ND	ND
formation of ammonia from peptone	+	-	ND	+	ND	ND	ND
H_2_S formation	-	-	-	ND	-	ND	ND
phosphatase activity	+	ND	ND	+	ND	ND	ND
Hydrolysis:							
starch	-	-	+	-	+		+
casein	+	+	ND	+	+		ND
gelatin	-	+	+	+	+		+
Carbon utilization:							
citrates	+	ND	ND	+	ND	ND	-
glycerin	+	-	-	ND	-	ND	ND
erythritol	-	ND	ND	ND	-	ND	ND
D-arabinose	-	ND	ND	ND	ND	ND	ND
L-arabinose	+	-	-	+	ND	ND	+
D-ribose	+	ND	ND	ND	ND	ND	ND
D-xylose	+	-	-	+	+	ND	+
L-xylose	-	ND	ND	ND	ND	ND	ND
D-adonitol	-	ND	ND	ND	ND	ND	ND
methyl-βD-xylopyranoside	-	ND	ND	ND	ND	ND	ND
D-galactose	+	-	-	+	+	ND	-
D-glucose	+	ND	ND	+	+	ND	-
D-fructose	+	ND	ND	+	-	ND	-
D-mannose	+	+	+	+	ND	ND	ND
D-sorbose	-	ND	ND	ND	ND	ND	ND
L-rhamnose	-	ND	ND	ND	ND	ND	+
dulcitol	-	ND	ND	ND	ND	ND	ND
inositol	+	ND	ND	+	ND	+	ND
D-mannitol	+	ND	ND	+	+	ND	ND
D-sorbitol	+	ND	ND	+	ND	ND	ND
methyl-αD-mannopyranoside	-	ND	ND	ND	ND	ND	ND
methyl-αD -glucopyranoside	-	ND	ND	ND	ND	ND	ND
N-cetylglucosamine	+	ND	ND	ND	+	ND	ND
amygdalin	-	ND	ND	ND	ND	ND	ND
arbutin	-	ND	ND	ND	ND	ND	ND
esculin (iron citrate)	+	-	-	ND	ND	ND	ND
salicin	-	-	-	ND	+	ND	ND
D-cellobiose	-	-	-	ND	+	ND	+
D-maltose	+	+	+	+	+	ND	+
D-lactose (bovine)	-	ND	ND	+	+	ND	+
D-melibioses	-	ND	ND	ND	ND	ND	ND
D-sucrose	+	-	-	+	ND	ND	-
D-trehalose	-	+	+	-	ND	+	ND
inulin	-	ND	ND	+	ND	ND	ND
D-melecytosis	-	ND	ND	-	ND	ND	ND
D-raffinose	-	ND	ND	-	ND	ND	ND
glycogen	-	ND	ND	ND	ND	ND	ND
xylitol	+	ND	ND	ND	ND	ND	ND
gentiobioses	-	ND	ND	ND	ND	ND	ND
D-turanose	-	ND	ND	ND	ND	ND	ND
D-lyxoses	+	ND	ND	ND	ND	ND	ND
D-tagatose	-	ND	ND	ND	ND	ND	ND
D-fucose	-	ND	ND	ND	ND	ND	ND
L-fucose	-	ND	ND	ND	ND	ND	ND
D-arabit	+	ND	ND	ND	ND	ND	ND
L-arabit	-	ND	ND	ND	ND	ND	ND
potassium gluconate	-	ND	ND	ND	ND	ND	ND
potassium 2-ketogluconate	+	ND	ND	ND	ND	ND	ND
potassium 5-ketogluconate	-	ND	ND	ND	ND	ND	ND

Note: -, no sign; +, sign found; ND, signs not found in the literature.

**Table 2:** The results of the antibacterial activity of the aboriginal pigment-forming strain of the Belgorod region.

№ ∏/∏	Test Cultures	JF4
1.	*Pseudomonas aeruginosa* ML4262	–
2.	*P. putida* KT2442	+
3.	*P. protegens* 38a	–
4.	*P. chlororaphis* PCL1391	–
5.	*P. fluorescens* B 849	+
6.	*P. caryophylli* BKM 1290	–
7.	*Escherichia coli* S	–
8.	*E. coli* B	–
9.	*Erwinia herbicola* ATCC 27155	–
10.	*Alcaligenes faecalis* BKM 1518	–
11.	*Bacillus cereus* GA5T	–
12.	*B. weihnestephanensis* KBA4	–
13.	*B. subtilis*	–
14.	*B. shaericus* BKM B509-1	–
15.	*B. thuringiensis* ATCC 35646	–
16.	*B. flexsus*	–
17.	*Micrococcus luteus* B1891	+
18.	*M. roseus* B1236	–
19.	St35	–
20.	*Arthrobacter* sp. B52	–
21.	*Deinococcus radiodurans*	–
22.	*Achromobacter ruhlandii* B-1330	+

Note: −, lack of antibacterial activity; +, antibacterial activity is present; the numbers describe the diameter of the zone of inhibition of the test culture.

**Table 3:** Resistance of the strain of the indigenous pigment-forming strain of the Belgorod region to antibiotics.

Antibiotic	Concentration	JF4
Amikacin	30 μg	+++
Amoxicillin	20 μg	++
Ampicillin	10 μg	–
Bacitracin	10 units	+
Bacitracin	0.04 units	–
Benzylpenicillin	10 units	–
Vancomycin	30 μg	+
Gentamicin	10 μg	+
Gentamicin	120 μg	+++
Imipenem	10 μg	–
Kanamycin	30 μg	++
Carbenicillin	25 μg	++
Carbenicillin	100 μg	++
Levomycin	30 μg	++
Lincomycin	15 μg	+
Meropenem	10 μg	–
Nalidixic acid	30 μg	+++
Neomycin	30 μg	+
Nystatin	80 units	+
Novobiocin		+
Norfloxacin	10 μg	+
Oxacillin	10 μg	–
Oxacillin	1 μg	–
Ofloxacin	5 μg	+++
Piperacillin	100 μg	+++
Polimxin	300 units	+
Rifampicin	5 μg	+
Streptomycin	30 μg	+
Streptomycin	300 μg	+ +
Tetracycline	30 μg	+
Tobralicin	10 μg	+++
Trimethoprim/Sulfamethoxazole	1.25/23.75 μg	+++
Furazolidone	300 μg	+++
Cefazolin	30 μg	–
Cefotaxin	30 μg	+++
Ceftazidine	30 μg	–
Ciprofloxacin	5 μg	+++
Erythromycin	15 μg	+

Note: −, antibiotic resistance; (+)–(+ + +), antibiotic sensitivity, according the size of the clearing zone around the antibiotic disc.

## Data Availability

The data presented in this study are available on request from the corresponding author.
